# The prevalences of *Neisseria gonorrhoeae and Chlamydia trachomatis* infections among female sex workers in China

**DOI:** 10.1186/1471-2458-13-121

**Published:** 2013-02-08

**Authors:** Xiang-Sheng Chen, Yue-Ping Yin, Guo-Jun Liang, Qian-Qiu Wang, Ning Jiang, Qiao Liu, Geng-Feng Fu, Bin Yang, Yu-Jiao Zhou, Mei-Qin Shi, Baoxi Wang

**Affiliations:** 1 National Center for STD Control, Chinese Academy of Medical Sciences and Peking Union Medical College Institute of Dermatology, Nanjing, China; 2 Hainan Provincial Institute of Department, Haikou, China; 3 Jiangsu Provincial Center for Disease Control and Prevention, Nanjing, China; 4 Guangdong Provincial Center for Skin Diseases and STD Control, Guangzhou, China; 5 Guangxi Center for Diseases Control and Prevention, Nanning, China

**Keywords:** Sexually transmitted infections, Prevalence, Female sex worker, China

## Abstract

**Background:**

Sexually transmitted infections (STIs) have become a major public health problem among female sex workers (FSWs) in China. There have been many studies on prevalences of HIV and syphilis but the data about *Neisseria gonorrhoeae* (NG) and *Chlamydia trachomatis* (CT) infections are limited in this population in China.

**Methods:**

A cross-sectional study was performed among FSWs recruited from different types of venues in 8 cities in China. An interview with questionnaire was conducted, followed by collection of a blood and cervical swab specimens for tests of HIV, syphilis, NG and CT infections.

**Results:**

A total of 3,099 FSWs were included in the study. The overall prevalence rates of HIV, syphilis, NG and CT were 0.26%, 6.45%, 5.91% and 17.30%, respectively. Being a FSW from low-tier venue (adjusted odds ratios [AOR]=1.39) had higher risk and being age of ≥ 21 years (AOR=0.60 for 21–25 years; AOR=0.29 for 26–30 years; AOR=0.35 for 31 years or above) had lower risk for CT infection; and having CT infection was significantly associated with NG infection.

**Conclusions:**

The high STI prevalence rates found among FSWs, especially among FSWs in low-tier sex work venues, suggest that the comprehensive prevention and control programs including not only behavioral interventions but also screening and medical care are needed to meet the needs of this population.

## Background

Heterosexual contact has become the predominant mode of HIV transmission in China, indicating that 46.5% of the estimated 740,000 people living with HIV and AIDS (PLWA) in 2011 were due to transmission through heterosexual contacts [1]. Female sex workers (FSWs) are one of the key populations to facilitate increase of the HIV epidemic and likely to determine how fast the HIV epidemic will spread from high risk groups to the general population [2]. In the national HIV/AIDS surveillance system in China, periodic prevalence surveys of syphilis infection among FSWs have been integrated but current *Neisseria gonorrhoeae* (NG) and *Chlamydia trachomatis* (CT) surveillance activities concentrate mainly on passive case reporting. Similarly, there have been many studies on prevalences of HIV and syphilis infections in this population [3-5] but few on other sexually transmitted infections (STIs) including NG and CT [2,6].These two infections can not only cause serious health complications [7] but act as facilitating factors in transmission of HIV [8]. In the study described in this article, we sought to determine the frequency of HIV, syphilis, NG and CT infections in a sample of FSWs recruited from 4 provinces in China, and to identify significant predictors associated with CT and NG infections.

## Methods

### Study population

The study was conducted in eight cities of 4 provinces in the eastern and southern parts of China (Changzhou and Yangzhou in Jiangsu, Hezhou and Wuzhou in Guangxi, Jiangmen and Maoming in Guangdong, and Qionghai and Sanya in Hainan), which was a part of baseline surveys in a China Mega Project to evaluate the impact of enhanced STI care on prevention of HIV infection among high risk groups in China. The details of study sites were reported previously [9]. Between June and September 2009, venues where FSWs solicited clients were mapped. The venues were classified into three subgroups, i.e. high-, middle- and low-class venues (HV, MV and LV). High-class venues included karaoke bars, or hotels; middle-class venues included hair salons or barber shops, massage parlors, foot bathing shops, roadside shops, guesthouses, or roadside restaurants; and low-class venues included street or other public outdoor places. FSWs who solicited clients in HV, MV and LV were named HV-, MV- and LV-based FSWs, respectively. A convenience sampling method was used to recruit FSWs by outreach workers. Participant eligibility requirements included age ≧15 years (to encompass FSWs representative of those at risk for STIs); ability to give consent; and having provided commercial sex in sex-work venues or rented apartments for money or goods within the previous three months.

### Data and specimen collection

All eligible FSWs were requested to participate in the study after receiving a brief description of the purpose and procedure of the study. Site staff secured verbal consent from subjects to have blood collected for free HIV and syphilis testing and cervical swab collected for free NG and CT testing, and to be confidentially interviewed with an anonymous and structured questionnaire by the trained outreach workers. All participants who provided specimens were informed of their test results by an outreach team member when the results were available (normally in a week for syphilis, a few week for HIV and 2–3 months for NG and CT). Participants with positive tests received counseling messages and were referred to designated clinics or disease control centres for further evaluation and possible treatment according to the national guidelines. A small gift priced at 30 yuan (around 5 US dollars) as an incentive was given to those women who agreed to participate in the survey. The study protocols were reviewed and approved by the Medical Ethics Committee of the Chinese Academy of Medical Sciences Institute of Dermatology and National Center for STD Control in Nanjing.

### Laboratory tests

Serological tests for detection of antibodies to HIV and syphilis and definitions of prevalent cases were reported previously [9,10]. Cervical specimens were evaluated at the National STD Reference Laboratory at Nanjing for NG and CT using polymerase chain reaction (PCR) based on Roche Amplicor assay (Roche Diagnostic Systems, Indianapolis, IN), as recommended by the manufacturer’s instructions. For calculating the prevalence, CT and NG infection were defined as having a positive PCR.

### Statistical analysis

A total of 3,099 FSWs who provided both blood and cervical swabs are included in the data analysis. Data were analyzed using SPSS (version 18.0 for Windows; SPSS Inc., Chicago, IL) and MedCalc (version 7.4 for Windows; Frank Schoonjans, Belgium). Outcome variables include prevalence rates and their 95% confidence intervals (CIs). Univariate analysis was used to determine association between characteristics and infection, and odds ratio (OR)was calculated. Factors with significance level of p<0.10 were included in multivariate logistic regression model to explore the association of indicators with acquisition of the infection. Adjusted odds ratio (AOR) and its 95% CI were estimated. Values of p<0.05 were considered statistically significant.

## Results

### Socio-demographic and behavioral characteristics

Each of the four study’s provinces accounted for about a quarter of the study sample size (759, 747, 795 and 798 from Jiangsu, Guangdong, Hainan and Guangxi, respectively). More FSWs in Hainan (22.4%) and Guangxi (18.9%) were of non-Han ethnicity than other two provinces (less than 8%). Among the 3,099 FSW participants, the mean age was 26.1 years (standard deviation [SD], 6.5; range, 15–53 years). About twenty percent of the participants were local registered residents, 21.4% were from other parts of the study province, and 58.3% were from outside of the province. Eighty percent had education level of secondary school or below, and 36.0% were currently married. Less than one-fifth (19.7%, 609/3099) of the FSWs currently worked in low-tier sex venues, and 41.9% (1298/3099) and 38.5% (1192/3099) were MV- and HV-based FSWs, respectively. The mean age of LV-based FSWs (28.3, SD 8.3 years) was significantly higher than that in MV- (26.5, SD 6.3, P<0.001) or HV-based FSWs (24.5 SD 5.2 years, P<0.001). Half (1611/3099) of the participants reported consistent use of a condom during the previous month and 74% reported use of a condom in the most recent commercial sex. Less than 5% of the FSWs reported having used an illicit drug.

### Prevalence of infection

The overall prevalence rates of HIV, syphilis, NG and CT in the study participants were 0.26 (95% CI, 0.13–0.51%), 6.45% (95% CI, 5.64–7.37%), 5.91% (5.13–6.80%) and 17.30 (95% CI, 16.01–18.67%), respectively. More than one-fifth (21.5%) of the FSWs were infected with either NG or CT. More than 28% (52/183) of FSWs with NG were co-infected with CT. As shown in Table 1, compared with that in age group of 15–20 years, older age groups had a significantly lower prevalence of NG or CT. A higher prevalence of CT was found in FSWs who never married. Infection of NG was found to be associated with CT infection.

**Table 1:** **Prevalence and odds of *C. trachomatis *and *N. gonorrhoeae *infections by socio-demographic and behavioral characteristics and other STIs**

**Factor**	**Sample size**	**Gonorrhoea**			**Chlamydia**		
		**%**	**OR (95% CI)**		**%**	**OR (95% CI)**	
FSW category							
High-tier	1192	5.54	Reference	—	16.36	Reference	—
Middle-tier	1298	5.24	0.94 (0.67–1.34)	0.74	16.87	1.04 (0.84–1.28)	0.73
Low-tier	609	8.04	1.49 (1.02–2.19)	0.04	20.03	1.28 (1.00–1.65)	0.05
Age group (years)							
15–20	617	8.10	Reference	—	27.88	Reference	—
21–25	1149	5.05	0.60 (0.41–0.89)	0.01	18.53	0.59 (0.47–0.74)	<0.001
26–30	648	4.94	0.59 (0.37–0.93)	0.02	10.03	0.29 (0.21–0.39)	<0.001
>30	685	6.28	0.76 (0.50–1.16)	0.20	12.55	0.37 (0.28–0.49)	<0.001
Geographic area							
Other provinces	2301	5.43	Reference	—	16.30	Reference	—
Guangxi	798	7.27	1.36 (0.99–1.88)	0.06	20.18	1.30 (1.06–1.59)	0.01
Education level							
Primary school or below	460	7.17	Reference	—	14.56	Reference	—
Secondary school	2074	5.59	0.77 (0.51–1.14)	0.19	17.84	1.27 (0.96–1.69)	0.09
High school or above	562	6.05	0.83 (0.51–1.37)	0.47	17.61	1.25 (0.89–1.76)	0.19
Marital status							
Never married	1890	5.38	Reference	—	19.79	Reference	—
Married	1113	6.65	1.25 (0.92–1.70)	0.15	13.57	0.64 (0.52–0.78)	<0.001
Divorced	87	5.75	1.07 (0.42–2.70)	0.88	11.49	0.53 (0.27–1.03)	0.06
Consistent condom use in past month							
Yes	1611	6.33	Reference	—	16.57	Reference	—
No	1488	5.44	0.85 (0.63–1.15)	0.29	17.08	1.11 (0.92–1.34)	0.27
Other STIs							
Syphilis	200	9.00	1.64 (0.99–2.73)	0.06	14.00	0.77 (0.51–1.15)	0.20
Gonorrhoea	183	—	—	—	28.41	1.99 (1.43–2.79)	<0.001
Chlamydia	536	9.70	1.99 (1.43–2.79)	<0.001	—	—	—

OR: odds ratio; CI: confidence interval.

### Risk factors of infection

In the multivariate analyses, the following factors were found to be significantly associated with CT infection at significance level of P<0.05 (Table 2): working in low-class sex venues (AOR 1.39, 95% CI 1.07–1.81, as compared with working in high-tier venues) and testing NG-positive (AOR 1.91, 95% CI 1.35–2.70, as compared with testing NG-negative) had higher risk, and being 21 years or above (AOR 0.60, 95% CI 0.48–0.76 for being 21–25 years; AOR 0.29, 95% CI 0.21–0.40 for being 26–30 years; AOR 0.35, 95% CI 0.26–0.47 for being 31 years or above, as compared with being 15–20 years in age) had lower risk for CT infection.

**Table 2:** **Factors associated With *C. trachomatis *and *N. gonorrhoeae *infections: results from multivariate logistic regression analysis**

**Independent factor**	**Gonorrhoea**	**Chlamydia**
Working in low-tier venues^*a*^	—	1.39 (1.07–1.81)*
Being 21–25 years old^*b*^	—	0.60 (0.48–0.76)**
Being 26–30 years old^*b*^	—	0.29 (0.21–0.40)**
Being 31 years or older^*b*^	—	0.35 (0.26–0.47)**
Having gonorrhoea^*c*^		1.91 (1.35–2.70)**
Having chlamydia^d^	1.99 (1.43–2.79)**	—

* P<0.05; ** P<0.01.

^*a*^ Reference group was FSWs working in high-tier venues.

^*b*^ Reference group was FSWs aged 20 years or younger.

^*c*^ Reference group was FSWs having not gonorrhoea.

^*d*^ Reference group was FSWs having not chlamydia.

## Discussion

To our knowledge, this study was a cross-sectional survey with biggest sample size of FSWs recruited from different categories of sex venues in multiple provinces in China. Our study finding that there was higher prevalence of chlamydial infection in LV-based FSWs is in accord with previous studies [2], and high prevalences of syphilis and HIV infections among this population and differences in these two infections between FSWs recruited from different categories of sex work venues were reported elsewhere [9,10]. In China, FSWs who solicit on streets or other outdoor places (freelance FSWs) and conduct commercial sex in rental houses are a special segment of the FSW population with more vulnerable nature in terms of socio-demographic characteristics, organization of sex work, employment and economic status, and relationship with clients [11,12]. Previous studies have shown that the FSWs working in low-class sex venues tended to be older in age, have high turnovers of sexual clients due to a low pay, but infrequently used condoms due to extra pay for unsafe sex [13]. The freelance FSWs either independently solicit clients on streets or find clients through nearby construction sites and factories [14]. They usually earn far less than what FSWs in higher-class sex venues earn but engage in riskier behaviors when having sex with both commercial and regular non-paying partners [12-14] and are highly stigmatized and marginalized by society. In addition, these FSWs may have poorer access to health information and health care services. High prevalence of NG or CT infection was found among FSWs, particuarly LV-based FSWs in the current study. Considering the synergistic effect of STIs on HIV infectivity and susceptibility [8], effective control of NG and CT in this population is not only important for avoiding the complications caused by these infections but also for preventing the transmission of HIV infection. A high co-infection of CT among FSWs infected with NG further supports the presumptive treatment of the patients with NG for CT.

Regarding sexual behaviors, self-reported condom use at last commercial sex was reported by 74.0% and consistent use of a condom during the previous month was reported by 52.0%. Unexpected was that the rates of condom use were not associated with NG or CT infections. This may be due, in part, to an upward bias in self-reported condom use rates [15]. Older age, which was usually associated with longer duration of working in sex trade, has also been identified as a risk factor for syphilis in previous studies among FSWs in China and other countries [16,17]. However, younger age is a significant risk factor for CT infection in the current study. A possible explanation is that young FSWs have more sexual activity, and less knowledge and experience with STI prevention. As well, younger women may have increased susceptibility to some STIs due to cervical ectopy following sexual initiation [18,19] or because of less likelihood of acquired protective immunity from previous STI exposure [20]. Previous study reported that the probability of incident chlamydial infection was inversely related to duration of prostitution [21]. Although the prevalence of CT infection among FSWs in low-class sex venues with higher risk behaviors was higher than that in middle- or high-class ones, the difference in the prevalence rates among LV-, MV- and HV-based FSWs was not as much as that for syphilis [9]. Previous study reported no difference in CT prevalence between establishment- and street-based FSWs [2]. Therefore, the likelihood of CT infection among FSWs may be not only related to the current risk behaviors but also the previous exposure to the infection which may be relevant to stimulation of protective immunity. In light of these findings and hypothesis further studies is warranted.

As FSWs are one of the important populations to drive the STI epidemic and probably the bridge population for the heterosexual transmission of STIs, the findings of this study have a number of important implications. First, this study shows substantial prevalences of NG and CT infections among FSWs and much higher prevalences of the infections among FSWs at low-class sex venues. The patterns of infections have served as a call for action to draw further attention to control the critical disease burden of CT and NG among this population and suggested the importance of including NG and CT prevalence surveys in the current surveillance program and prioritizing the FSWs at low-tier venues in the current program for intervention. Second, this study indicates the association of young age with CT infection, which further supports the concept of protective immunity to the recurrent infections although more studies are needed. Based on these findings, it is important to consider the potential protective immunity related to age and duration of prostitution when we use CT or NC prevalence as a proxy to indicate risk behaviours among high-risk groups. However, further biological and epidemiological studies are needed.

Several limitations of this study should be considered in the interpretation of the results. First, study participants were not randomly recruited, so they may not be an accurate representation of the target study population. Second, as prostitution is illegal in China, some FSWs (ranging from 10% to 40% and varying between different types of venues) refused participating in the study, resulting in potential selection bias. Third, our results may also be affected by a self-reporting bias, particularly those related to sexual behaviours.

## Conclusion

The high NG and CT prevalence rates found among FSWs in China, especially among LV-based FSWs, suggest that it is essential that comprehensive prevention and control programs including not only behavioral interventions but also screening and medical care are needed to meet the needs of this high-risk population.

## Competing interests

The authors declare that they have no competing interests.

## Authors’ contributions

XSC, YPY, QQW, GJL, NJ and BW conceived the study and participated in study design. QL, XPH, BY, YJZ, DLP, GFF and LGY participated in implementation of the study in sites and data collection. MQS coordinated the laboratory testing of the infections and helped to interpret the data. NJ and XSC performed the statistical analysis. XSC prepared the manuscript with assistance of YPY, QQW, NJ and BW. All authors read and approved the final manuscript.

## Pre-publication history

The pre-publication history for this paper can be accessed here:

http://www.biomedcentral.com/1471-2458/13/121/prepub
